# Divergent evolution of colony‐level metabolic scaling in ants

**DOI:** 10.1111/1365-2656.70055

**Published:** 2025-05-07

**Authors:** Pedro A. C. L. Pequeno, Douglas S. Glazier

**Affiliations:** ^1^ Natural Resources Program Federal University of Roraima Boa Vista Brazil; ^2^ Department of Biology Juniata College Huntingdon Pennsylvania USA

**Keywords:** caste polymorphism, eusocial insects, foraging, metabolic level, metabolic scaling, trophic niche

## Abstract

1. Metabolic scaling—the relationship between organismal metabolic rate (*R*) and body mass (*M*)—is an important property of life. In general, this relationship has been summarized by the scaling function, *R* = *aM* ^*b*^.
2. Both the scaling elevation (*a*) and the scaling exponent (*b*) have been shown to diverge among taxa and ecological groups. However, it is unclear whether this ecological divergence observed in unitary organisms also occurs at higher levels of biological organization, such as eusocial colonies.
3. We used the published literature to assemble the estimates of the metabolic rate of active colonies and their mass for 51 species of ants, along with three ecologically important traits with available data: trophic level (herbivorous to predaceous), foraging coordination level (solitary to trunk trail) and caste polymorphism (polymorphic vs. monomorphic).
4. Interspecific colony metabolic scaling was steeper (higher *b*) in species occupying higher trophic levels and in species with polymorphic versus monomorphic workers. Species occupying higher trophic levels also had a higher metabolic level (higher *a*).
5. These findings are consistent with divergent selection on colony‐level metabolic scaling. We conclude that the ecological dependence of metabolic scaling has evolved across levels of biological organization and should be explicitly considered by both metabolic and social evolution theories.

## Graphical abstract

The study shows for the first time that the metabolic scaling of active ant colonies varies as a function of trophic level and worker polymorphism. Therefore, the ecological divergence of metabolic scaling observed in unitary organisms occurs across levels of biological organization.

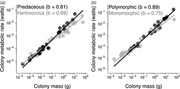

## 1 INTRODUCTION

All organisms need energy to survive and reproduce. In general, the rate of energy use or metabolic rate (*R*) increases with body mass (*M*), which can often be represented by the power function, *R* = *aM* ^*b*^. However, metabolic rate usually increases more slowly than does increasing body mass (hypometric scaling, *b* < 1), with *b* varying mostly between 0.5 and 1.0 (Glazier, 2022; Harrison et al., 2022; White & Kearney, 2013), sometimes in association with metabolic level (the scaling elevation *a*) (Glazier, 2010, 2014). Despite the ubiquity of hypometric metabolic scaling, its causes remain uncertain (Glazier, 2022; Harrison et al., 2022; White & Kearney, 2013). In unitary organisms, variation in scaling patterns has been shown to correlate with various ecological factors, suggesting adaptive divergence (García‐Gómez et al., 2024; Glazier, 2006; Killen et al., 2007; Pequeno et al., 2017). However, it is not clear whether this ecological divergence also applies to higher levels of biological organization, such as colonies of eusocial animals (Fewell & Harrison, 2016; Glazier, 2014; Harrison et al., 2022; Waters, 2014). Therefore, comparative analyses of ecologically and socially diverse taxa may help discriminate between alternative hypotheses on metabolic scaling and reveal common rules across levels of biological organization.

Major theories of metabolic scaling posit the importance of body geometry (e.g. surfaces or networks that transport matter and energy), body composition (e.g. tissues and cells with different maintenance costs) and resource demand (e.g. costs of behaviour and production), whose effects can interact (Glazier, 2014; Harrison et al., 2022; White & Kearney, 2013). For instance, in active, unitary organisms, the scaling slope *b* often increases from 2/3 to 1 as metabolic level increases, consistent with a shift from metabolic limitation by body surfaces available for resource flow (which scale as mass^2/3^) to limitation by the resource demand of volume‐filling tissue such as muscles (which is proportional to mass) (García‐Gómez et al., 2024; Glazier, 2010, 2014). Thus, the scaling slope can respond to resource demand (metabolic level) within boundaries set by body geometry and composition. In turn, metabolic level may reflect ecological adaptations. For instance, when active, more athletic species tend to have higher metabolic level and scaling slope (Killen et al., 2007; Weibel et al., 2004), as do fast‐growing species (Glazier, 2006). Ecological factors such as food abundance and quality can also affect metabolic level (McNab, 1986; Mueller & Diamond, 2001; Naya et al., 2013; Sabat et al., 2009) and/or the scaling slope (Pequeno et al., 2017). Furthermore, the boundaries of the scaling slope may change if body shape and/or the proportion of metabolically costly tissue also changes with body mass (Glazier, 2010, 2014).

Eusocial insects such as ants and termites dominate the animal biomass of terrestrial ecosystems, rivalled only by humans and livestock (Tuma et al., 2020). This dominance reflects their cooperative colonies characterized by reproductive division of labour (queen–worker dimorphism), sometimes with further division among sterile castes (Hölldobler & Wilson, 2009). When queen–worker dimorphism is genetically fixed, colonies are analogous to multicellular organisms with germ–soma separation. Thus, they can be considered a higher level of individuality or ‘superorganisms’ (Boomsma & Gawne, 2018; Hölldobler & Wilson, 2009). Indeed, the interspecific metabolic scaling of active eusocial colonies has been found to resemble that of unitary organisms (Hou et al., 2010; Mason et al., 2015; Shik et al., 2012). However, little is known about the degree of ecological divergence in colony metabolic scaling (Shik et al., 2014). Furthermore, colonies grow by adding separate, movable individuals, whose total surface area should scale proportionately to total volume (*b* = 1) rather than hypometrically (*b* = 2/3) as in unitary organisms. Therefore, colony geometry is unlikely to explain hypometric metabolic scaling (Glazier, 2014; Harrison et al., 2022; Negroni & LeBoeuf, 2023). Rather, factors affecting colony composition and/or resource demand may play this role and thus underlie divergent scaling relationships.

Ants are the most diverse and best studied clade containing only eusocial species (Hölldobler & Wilson, 2009). Metabolic hypometry in active ant colonies has been linked to larger colonies having larger individuals (including larger bodied castes), whose lower mass‐specific metabolic rate decreases colony respiration per gram (Shik, 2010; Shik et al., 2012). Also, larger colonies may have a larger proportion of inactive individuals (Waters et al., 2010, 2017) or greater insulation among individuals that reduces overall interaction rate (Toth et al., 2023). Likewise, increased specialization and division of labour in larger colonies may provide efficiency gains, thus decreasing mass‐specific respiration (Fewell & Harrison, 2016; Jaffé & Fonck, 1994; Negroni & LeBoeuf, 2023). Therefore, species traits affecting body size distribution, activity level and/or division of labour could affect colony metabolic level and/or metabolic scaling.

Ant diet varies across species from plant exudates to animal prey (Drager et al., 2023). Plant resources such as sugar‐rich exudates are more spatially predictable (Lanan, 2014), which may reduce the metabolic costs of foraging. Yet, the greater reliability of plant resources could favour higher production rates (Hölldobler & Wilson, 2009; Sibly & Brown, 2007), with workers using plant exudates to fuel higher activity levels and colony growth (Chinarelli et al., 2021; Grover et al., 2007). Likewise, ant foraging tactics vary from solitary foraging to collective foraging coordinated by pheromone trails (Beckers et al., 1989; Lanan, 2014). Although more coordinated foraging systems may depend on costly pheromones and/or brains whose cost increases metabolic rate, optimization of collective behaviour may increase task efficiency and reduce metabolic costs per individual (Coto & Traniello, 2022; Jaffé & Fonck, 1994). Alternatively, trail pheromones enhance competitive ability (Westermann et al., 2014), allowing higher production rates. Overall, then, trophic level and foraging coordination could either increase or decrease metabolic level and the scaling slope.

Moreover, approximately 13% of ant species are polymorphic, with workers varying in body size and/or shape and specializing in different tasks (Wills et al., 2018). Division of labour may reduce metabolic costs by increasing task efficiency and/or adding larger bodied castes that respire less per gram (Coto & Traniello, 2022; Fewell & Harrison, 2016; Waters, 2014), but may also require costly mechanisms of coordination such as chemical signalling (Jaffé & Fonck, 1994; McCarthy & Enquist, 2005). Furthermore, caste polymorphism may improve resource acquisition by allowing faster detection, processing and/or retrieval (Feinerman & Traniello, 2016; La Richelière et al., 2022; McGlynn & Owen, 2002; Pequeno, 2024). If so, polymorphic species may maintain higher activity and/or production rates. Hence, as with trophic level and foraging coordination, caste polymorphism could either increase or decrease metabolic level and, consequently, the scaling slope.

Here, we investigated these issues by taking advantage of the increasing availability of global compilations of ant trait data. We assembled a comparative dataset on 51 ant species and tested whether colony‐level metabolic scaling has shifted with trophic level, foraging coordination level and caste polymorphism, as considered by the above hypotheses.

## 2 MATERIALS AND METHODS

### 2.1 Data assembly

Metabolic rates of active ant colonies and their masses were obtained from published data compilations (Shik et al., 2012, 2014), references therein and studies citing those references in Google Scholar. Then, data on trophic level, foraging coordination level and caste polymorphism of those species were searched for in published data compilations on those traits (see below). Only species for which all analysed variables were available were used. Only one study not included in the compilations met this criterion (Mason et al., 2015). Data provided in the compilations were compared to those in the original studies to guarantee they were correct. The combined dataset comprised 53 ant species in seven subfamilies, sampled by nine studies across 10 sites in five countries (Belgium, Panama, South Africa, United States and Venezuela) (Figure S1). However, only 51 species were analysed due to methodological issues (see below). All variables were averaged across colonies per species, with 1–25 colonies measured for metabolism per species (mean of 3.6). Phylogenetic relationships among the analysed species were manually coded in Newick format using a genus‐level tree as reference (AntWiki, 2024) (Figure S2). Within‐genus relationships were represented as polytomies, and branch lengths were standardized to the same value (one). Species names were checked for validity and updated as required according to an online database (AntWiki, 2024).

Metabolic rate was estimated from CO_2_ produced in respirometry chambers, but method details varied. For most species, colonies were collected in the field, had their natural composition and were measured using flow‐through respirometry. For some species, colonies were reared from queens for 1 year in the laboratory, had their composition set to an adult:brood ratio of 4:1 and/or were measured using closed‐system respirometry. All colonies had food ad libitum prior to metabolic measurements, but colony maintenance conditions varied (and this was accounted for in the analysis; see below). For a few species, the largest colonies were too large for the respirometry chamber, so colonies were fragmented into two or three subcolonies, which were averaged to represent the species. All colonies were considered active, based on direct observation reported by some of the original studies and on the fact that, where measured, whole colonies tend to have a higher mass‐specific metabolic rate than isolated worker groups (Fewell & Harrison, 2016).

Most colony metabolic rates were provided at 25°C, but some were provided at 30°C. Accordingly, all values were standardized to 25°C assuming *Q*_10_ = 2.00, as in previous interspecific analyses of ant colony metabolic scaling (Shik et al., 2012, 2014). Although *Q*_10_ is variable, estimates for ant species cluster near 2.0 (Willot et al., 2023), so we kept this value for consistency with the literature. Furthermore, the possibility of geographic variation in Q_10_ and its impact on colony metabolic rate was accounted for in the analysis (see below). All metabolic measurements were expressed in Watts.

Colonies used for respirometry were weighed to determine their mass. Wet mass was reported for most species, but only dry mass was reported for some. Because we were interested in living colonies, we considered the former. We used species for which both measurements were available to regress wet colony mass (*Y*) on dry colony mass (*X*) (both log_10_‐transformed) and then predicted wet colony mass for those species for which only dry mass was reported (*n* = 27, log_10_ *Y* = −2.80 + 1.22 log_10_ *X*, *r* ^2^ = 0.99, *p* < 0.001).

Trophic level was obtained from a global data compilation including 393 species (Drager et al., 2023). The average trophic level of ant species was estimated from nitrogen stable isotope ratios by assuming trophic level increases with the difference between the ratios of the focal ant and of a baseline (a co‐occurring primary producer). In ants, trophic level ranges between 1 (herbivores) and 5 (top predators) (Drager et al., 2023). Direct estimates of trophic level were available for 13 of the analysed species, so we used genera means for the remaining species. This assumes that congeneric species tend to have similar trophic levels, which is supported by the high phylogenetic signal of this trait (Pagel's *λ* = 0.74; Drager et al., 2023). For *Nylanderia guatemalensis*, the mean trophic level of its former genus (*Paratrechina*) was used, as there was no data for *Nylanderia*.

Foraging coordination level was assigned based on published data compilations on ant foraging tactics, covering over 400 species (Beckers et al., 1989; Lanan, 2014). Ant foraging can be ranked by increasing coordination among foragers. For the analysed species, the following levels can be recognized: (1) solitary foraging: foragers search for food and collect it individually; (2) tandem running: a forager that returns to the nest after successfully finding a resource guides a single nestmate to that resource; (3) group recruitment: returning foragers guide groups of nestmates to detected resources; (4) mass recruitment: returning foragers lay short‐lived pheromone trails from the food to the nest, which then guide nestmates to the food; (5) trunk trails: long‐lived trails are laid between stable food sources and the nest that both guide foragers and serve as starting points for new trails. These groups correlate with other ecological traits such as colony size and resource spatiotemporal distribution (Beckers et al., 1989; Lanan, 2014), supporting their biological meaning. Hence, foraging coordination level was coded as an ordinal variable: solitary foraging (1), tandem running (2), group recruitment (3), mass recruitment (4) and trunk trail (5).

Caste polymorphism was assigned based on a global data compilation of 8890 species (La Richelière et al., 2022). These authors classified each species as either polymorphic (1) or monomorphic (0), defining polymorphism as any interindividual variation in size or head‐to‐body allometry reported in the primary literature, either continuous or discrete variation. Species for which there were no published data were assigned the same category as congeneric species (La Richelière et al., 2022). Here, 47 species could have their polymorphism directly assigned from the compilation. For the remaining six species (*Brachymyrmex* sp., *Crematogaster* sp., *Leptothorax unifasciatus* and three unidentified *Solenopsis* spp.), we obtained the means of their respective genera as an estimate of the probability of being polymorphic given the known proportion of polymorphism in the genus. Species in genera where this probability was larger than 0.5 were classified as polymorphic (1) and monomorphic otherwise (0).

Shik et al. (2014) showed that some fungus‐farming ant species tended to have a lower colony metabolic rate for a given colony mass compared to the remaining hunter–gatherer ants. However, they considered ant and fungus metabolism together in scaling analyses. In our dataset, seven species were fungus farmers, and the data allowed separating ant and fungus metabolism for five of them. Considering only the ant metabolism and mass of such species, fungus farmers followed the same colony metabolic scaling of the remaining species (Figure S3). Given this pattern and the small sample size of fungus farmers, we did not consider this variable in the current analysis. Thus, the dataset analysed contained 51 species, considering only the ant part of fungus farmers. This study did not require ethical approval.

### 2.2 Data analysis

To test the role of ecological and social traits in modifying the metabolic scaling of active ant colonies, we used linear mixed models accounting for phylogenetic autocorrelation. Whole‐colony metabolic rate was the response variable, whereas colony mass was the main predictor (both log_10_‐transformed). Furthermore, interaction terms between colony mass and each trait were included. Study identity and sampling site were included as random factors to account for methodological and geographic variation in metabolic rate, respectively. Three studies that used the same methods were grouped together, and site was coded as a nested random factor within study given that some studies sampled more than one site. Residual phylogenetic autocorrelation was given by Pagel's *λ*, which is robust to phylogenetic uncertainty (Molina‐Venegas & Rodríguez, 2017). We also assessed the potential for multicollinearity issues by computing all pairwise phylogenetic correlations between predictors, which were moderate to low (*r* ≤ 0.5; Table S1).

First, a full model was fitted including all pairwise interactions between colony mass and ecological traits. Then, unsupported interactions (*p* > 0.05) were excluded, and the model was fitted again to test for independent effects of ecological traits. We accessed model predictive power with the marginal *R* ^2^ ($R_{m}^{2}$) and the conditional *R* ^2^ ($R_{c}^{2}$), which measure variation explained by predictors alone and by predictors plus random factors, respectively. To visualize results, partial residuals were plotted against supported predictors, which control for the remaining terms in the model (Breheny & Burchett, 2017). To facilitate interpretation of shifts in metabolic scaling involving quantitative predictors, the final model was used to obtain the scaling exponent and its respective 95% confidence interval (CI_95%_) for groups defined as above and below the mean of that predictor, while keeping each group and all remaining variables at their own means (or their modes for binary variables). We did not categorize trophic level (e.g. herbivores vs. predators) in the analysis itself as categorizing continuous variables reduces statistical power, among other statistical issues (Beltran & Tarwater, 2024).

To determine whether shifts in metabolic scaling correlated with changes in metabolic level, two complementary measures of metabolic level were used (Glazier, 2010): the scaling intercept (predicted metabolic rate where log_10_‐colony mass equals zero, equivalent to 1 g), which standardizes colony mass but is intrinsically correlated with the scaling slope on a log scale; and the midpoint metabolic rate (predicted mass‐specific metabolic rate at the midpoint of the log colony mass range), which does not standardize colony mass but is independent of the scaling slope on log scale. For each trait affecting metabolic scaling, 95% confidence intervals were computed for both the intercept and midpoint estimates of contrasting groups of species. Groups were considered to have different metabolic levels if less than half of one side of the confidence intervals overlapped (equivalent to *p* < 0.05; Cumming, 2009).

All analyses were performed in R 4.2.2 (R Core Team, 2022), with the aid of packages ‘ape’ (Paradis et al., 2004), ‘caper’ (Orme et al., 2018), ‘nlme’ (Pinheiro et al., 2020), ‘MuMIn’ (Barton, 2019), ‘visreg’ (Breheny & Burchett, 2017), ‘AICcmodavg’ (Mazerolle, 2023) and ‘marginaleffects’ (Arel‐Bundock, 2024).

## 3 RESULTS

Colony metabolic rate varied from 0.004 mW in *Strumigenys brevicornis* to 73.8 mW in *Camponotus fulvopilosus*, with a phylogenetic signal of *λ* = 0.73 (95% CI = 0.01–1.00). Colony mass varied from 0.0002 g in *S. brevicornis* to 57.33 g in *Pachycondyla villosa*, with a phylogenetic signal of *λ* = 0.86 (95% CI = 0.06–1.00). Trophic level ranged from 1.66 in *Leptothorax unifasciatus* to 4.21 in *Gnamptogenys horni*, with *λ* = 1.00 (95% CI = 0.9–1.00). Foraging coordination level varied from 1 to 5 and averaged 3.4 (*λ* = 1.00, 95% CI = 0.94–1.00), while 37% of the 51 species analysed were polymorphic (*λ* = 1.00, 95% CI = 0.69–1.00).

**FIGURE 1:**
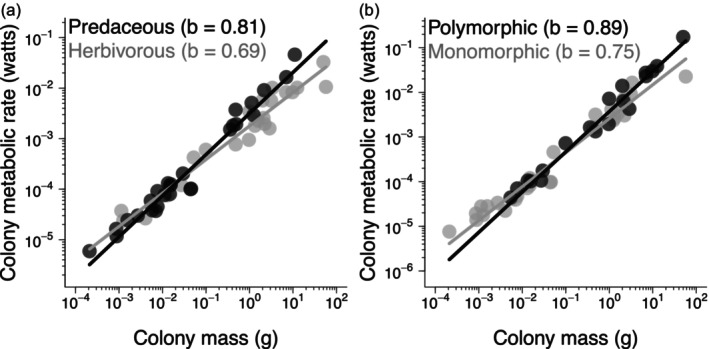
Predictors of colony metabolic rate in ants. Each point is one species (*n* = 51), lines are predictions of the final model (Table 1) and legends indicate species groups and their respective scaling exponents (*b*). Vertical axes are partial residuals, which represent variation in the response variable after accounting for remaining factors in the model. (a) Different scaling of colony metabolic rate between trophic levels. Species were grouped for visual convenience as ‘predaceous’ if above the mean trophic level of 2.91 and ‘herbivorous’ otherwise. (b) Different scaling of colony metabolic rate between polymorphic and monomorphic species.

The models on colony metabolic rate explained almost all the observed variability among species ($R_{m}^{2}=R_{c}^{2}=0.96$, Table 1; notice the small scatter of the data in Figure 1). The full model revealed no evidence for an interaction between colony mass and foraging coordination level (Table 1). However, there was support for an interaction between colony mass and trophic level and between colony mass and caste polymorphism (Table 1). The final model excluding non‐supported interactions confirmed these effects and further showed that foraging coordination level had no independent effect (Table 1).

**TABLE 1:** Linear mixed models of interspecific colony metabolic scaling in ants (*n* = 51 species).

Model	Predictor	Coefficient (95% CL)	*t*	*p*
Full	Intercept	−3.36 (−3.93, −2.80)	—	—
	Mass	0.30 (−0.14, 0.74)	1.387	0.175
	Trophic	0.23 (0.05, 0.42)	2.531	**0.016**
	Foraging	0.02 (−0.03, 0.08)	0.980	0.334
	Poly	0.15 (−0.03, 0.34)	1.663	0.106
	Mass × Trophic	0.14 (0.02, 0.28)	2.337	**0.026**
	Mass × Foraging	0.01 (−0.02, 0.04)	0.454	0.652
	Mass × Poly	0.13 (0.01, 0.25)	2.259	**0.031**
Final	Intercept	−3.34 (−3.90, −2.80)	—	—
	Mass	0.34 (−0.03, 0.73)	1.861	0.071
	Trophic	0.22 (0.04, 0.41)	2.517	**0.017**
	Foraging	0.02 (−0.02, 0.07)	0.877	0.387
	Poly	0.16 (−0.02, 0.34)	1.774	0.085
	Mass × Trophic	0.14 (0.02, 0.26)	2.365	**0.024**
	Mass × Poly	0.14 (0.03, 0.26)	2.509	**0.017**

*Note*: Colony metabolic rate and colony mass were log_10_‐transformed. The full model tested the three interactions between colony mass and ecological traits, whereas the final model excluded non‐significant interactions to test trait‐independent effects. Models accounted for residual phylogenetic autocorrelation (final model's Pagel *λ* = 0.41; 95% confidence limits: 0, 0.9). Study and sampling site were used as nested random factors to control methodological/geographic variation but did not explain any variation beyond predictors (for both models, $R_{m}^{2}=R_{c}^{2}=0.96$). 95% CL: 95% confidence limits. Bold numbers indicate statistical support (*p* < 0.05). Mass: Colony wet mass. Trophic: Trophic level. Foraging: Foraging coordination level. Poly: Caste polymorphism (0 for monomorphic, 1 for polymorphic).

**FIGURE 2:**
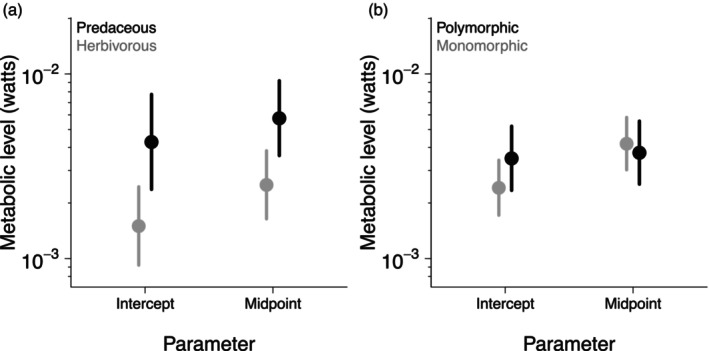
Alternative measures of metabolic level in relation to trophic level (a) (‘predaceous’ if above the mean trophic level, ‘herbivorous’ otherwise) and (b) caste polymorphism (polymorphic vs. monomorphic species), based on the final model (Table 1). Metabolic level was estimated for each species group using both the intercepts of scaling relationship (predicted metabolic rate where log_10_ colony mass was zero, equivalent to 1 g) and the midpoint (predicted mass‐specific metabolic rate at the midpoint of the colony mass range). Error bars are 95% confidence intervals.

Colony metabolic scaling was steeper for species with a higher trophic level (i.e. predominantly predaceous; Figure 1a) and with polymorphic castes (Figure 1b). By separating species as mostly herbivorous or predaceous (below and above the mean trophic level of 2.91, respectively), the scaling exponent was calculated as *b* = 0.69 (CI_95%_: 0.58–0.79) for the former group and *b* = 0.81 (CI_95%_: 0.74–0.89) for the latter. For caste polymorphism, we obtained *b* = 0.75 (CI_95%_: 0.68–0.82) for monomorphic species and *b* = 0.89 (CI_95%_: 0.79–1.00) for polymorphic species. Lastly, the two types of estimates of metabolic level increased with trophic level (Figure 2a) but were similar between polymorphic and monomorphic species (Figure 2b).

## 4 DISCUSSION

This study revealed that the metabolic rate of active ant colonies increased faster with colony mass across species from higher trophic levels and across polymorphic versus monomorphic species. Furthermore, the metabolic level also increased with trophic level. These findings refute suggestions of a single colony‐level metabolic scaling exponent in eusocial insects (Hou et al., 2010). Rather, metabolic scaling has seemingly adapted to divergent ecological pressures not only in unitary organisms (Glazier, 2022; Killen et al., 2010; Pequeno et al., 2017) but also in eusocial colonies.

We hypothesized that adaptation to abundant, reliable plant resources could either decrease foraging costs (Lanan, 2014) or sustain higher production rates in herbivorous species (Hölldobler & Wilson, 2009; Sibly & Brown, 2007). Hence, trophic level could either increase or decrease metabolic level. In turn, in active organisms, higher metabolic level could increase the scaling slope towards 1.0 due to the increasing influence of volume‐filling muscles on metabolic costs (García‐Gómez et al., 2024; Glazier, 2010, 2014). Indeed, active colonies of predaceous ant species had a higher metabolic level (Figure 2a) and steeper metabolic scaling (Figure 1b), consistent with higher foraging demands in predaceous species. In terrestrial vertebrates, predators tend to have larger home ranges and steeper scaling of home range sizes than herbivores (Schoener, 1971; Tamburello et al., 2015). This is because predators face increasing scarceness of suitable prey as they grow larger, whereas larger herbivores can find plant resources more easily. In turn, species with larger home ranges can have higher mass‐specific metabolic rates due to selection for increased muscular endurance (Muñoz‐Garcia & Williams, 2005). Seeking prey may also require more complicated decisions by a more costly brain (Azorsa et al., 2022). Hence, predaceous species might have evolved higher metabolic level and, consequently, steeper metabolic scaling to match their required foraging area. This assumes that colony metabolic rate is proportional to foraging area, as specified by some models of colony foraging (Jun et al., 2003). Indeed, predatory ants tend to forage more diffusely than herbivorous ants, consistent with the greater spatial unpredictability of animal resources (Hanisch et al., 2023; Lanan, 2014).

The steeper scaling of colony metabolic rate in polymorphic species (Figure 1b) seems incompatible with the idea that polymorphism reduces metabolic costs through higher task efficiency (Coto & Traniello, 2022; Fewell & Harrison, 2016; Negroni & LeBoeuf, 2023; Waters, 2014). Rather, this finding is more consistent with higher resource demand by more complex ant societies (Jaffé & Fonck, 1994), since higher demand can drive steeper metabolic scaling in unitary organisms (Glazier, 2006, 2010, 2014). For instance, caste specialization could increase overall metabolic costs by causing individuals to perform their tasks at a consistently high rate (Jaffé & Fonck, 1994; Sempo & Detrain, 2010). Also, polymorphic workers can have complementary niches, allowing the colony to forage under a wider environmental range (Wills et al., 2018). Larger workers can tolerate hotter and drier conditions or retrieve food items that smaller workers cannot. Therefore, polymorphic species may maintain higher foraging levels and faster colony growth, which are sustained by steeper colony‐level metabolic scaling. Some models of ant colony metabolism predict that polymorphic species should have a fitness advantage over monomorphic species when resources are scarce (Feinerman & Traniello, 2016). Therefore, caste polymorphism—and perhaps more generally, division of labour—may be a costly but effective strategy for higher resource acquisition (McCarthy & Enquist, 2005; Pequeno, 2024). Accordingly, polymorphic ant species tend to occur in warmer and drier regions of the world, where resources are presumably scarcer, and polymorphism may provide a competitive advantage (La Richelière et al., 2022). Also, in another major clade of eusocial insects, termites, sterile caste number is strongly related to the ability to forage outside the nest, consistent with polymorphism releasing species from local resource depletion (Pequeno, 2024).

Interestingly, the steeper metabolic scaling of active colonies of polymorphic versus monomorphic species did not reflect a higher metabolic level in polymorphic species (Figure 2b). This contrasts with the joint increase in metabolic level and scaling slope observed with trophic level (Figure 1a), which has also been observed in active, unitary organisms (García‐Gómez et al., 2024; Glazier, 2010, 2014). Yet, the scaling slope may become steeper independently of metabolic level if costly functions or structures also increase disproportionately with size, for example, increasing endothermy in growing vertebrates and increasing growth rate in larger prokaryotes (Glazier, 2010, 2014). Indeed, caste polymorphism occurred only in colonies weighing above ca. 10^−2^ g (Figure 1b; *r* = 0.5; Table S1), which might account for steeper metabolic scaling in polymorphic versus monomorphic species despite their similar metabolic levels.

The range of scaling exponents found here (*b* between 0.69 and 0.89) is within that found by intraspecific studies on ant colony metabolic scaling (Fewell & Harrison, 2016; Waters, 2014) and by interspecific studies comparing taxa of unitary organisms (Glazier, 2022; Harrison et al., 2022; White & Kearney, 2013). While this might suggest a common basis for metabolic scaling across levels of biological organization, it is also possible that various mechanisms operate in different contexts (Glazier, 2014). Also, while studies on ant colony metabolic scaling have focused on intraspecific variation (Fewell & Harrison, 2016), mechanisms accounting for interspecific patterns may differ. For instance, activity level can explain hypometric scaling of colony metabolic rate within species (Waters et al., 2010, 2017), but there is no obvious relationship between activity level (at least in terms of proportion of foragers) and colony size across species, although data are scarce (Nobua‐Behrmann et al., 2013; Tschinkel, 2011). Clearly, more data are required to better understand the proximate mechanisms underlying evolutionary patterns of colony metabolic scaling in eusocial animals.

Several limitations should be considered. First, colonies measured under laboratory conditions may not behave as they would under natural conditions, which may bias adaptive interpretations (Harrison et al., 2022). Second, ant colony size ranges from less than 10 individuals to hundreds of millions (Burchill & Moreau, 2016), but metabolic measurements are biased towards colonies no larger than hundreds of individuals (Shik et al., 2014). This is because respirometry of entire ant colonies is challenging, and even colonies not in the upper limit of the size range may need to be separated into smaller groups that can fit into measurement chambers (Mason et al., 2015). Third, while we found no effect of foraging coordination level on colony metabolic scaling, there were no data for swarm raiders such as army ants, which have one of the most coordinated foraging tactics (Beckers et al., 1989). Fourth, we classified species as monomorphic versus polymorphic due to data availability, but caste polymorphism varies quantitatively and may have more subtle effects. The role of polymorphism could be further investigated by measuring polymorphic species with very large colonies such as army ants, or by measuring polymorphism with morphometric data. More generally, adding more species should improve statistical power and the precision of estimates. Finally, our analysis cannot determine causality, and our interpretations rely on certain unmeasured variables such as colony foraging area. Eusocial insect colonies are amenable to experimental manipulation and may be used to test directly for ecological effects on metabolic scaling (Naug, 2024).

This study showed that the interspecific colony metabolic scaling of ants has diverged with trophic level and caste polymorphism. While the precise mechanisms involved require further study, these findings are consistent with divergent selection on colony‐level metabolism. We conclude that evolutionary divergence of metabolic scaling is not limited to unitary organisms but extends to eusocial colonies. Thus, theories on the causes and consequences of metabolic scaling and social evolution should account for such divergence both at the individual and colony levels.

## AUTHOR CONTRIBUTIONS

Pedro Aurélio Costa Lima Pequeno and Douglas S. Glazier conceived the ideas and designed methodology; Pedro Aurélio Costa Lima Pequeno collected and analysed the data and led the writing of the manuscript. Both authors contributed critically to the drafts and gave final approval for publication. Statement of inclusion: This study was a comparative analysis based on literature data from several continents. As such, there was no local data collection. However, the authors represent the continents contributing most data (the Americas) and encompass diverse backgrounds, reflecting the countries in which they are based: Brazil (PACLP) and the United States (DSG).

## CONFLICT OF INTEREST STATEMENT

We have no conflict of interest to declare.

## Supporting information


**Figure S1:** Geographic distribution of ant colonies measured for metabolic rate in this study.
**Figure S2:** Phylogenetic tree of the analyzed ant species.
**Figure S3:** Interspecific colony metabolic scaling of ants, with data for fungus‐farming species mixing the ant and fungus part of colonies (left; *n* = 53 species) and separating them (right; *n* = 51 species).
**Table S1:** Phylogenetic correlations (and their respective *p* values) among ant species traits used as predictors in the analyses of colony‐level metabolic scaling.

## Data Availability

Data available from the figshare repository https://doi.org/10.6084/m9.figshare.27207690.v2 (Pequeno & Glazier, 2025).
