# Supplementary material for: Divergent evolution of colony‐level metabolic scaling in ants
Source: J Anim Ecol. 2025 May 7;94(6):1285–93. doi: 10.1111/1365-2656.70055 (PMC12134421; doi:10.1111/1365-2656.70055)
Supplement: Supplementary file 1 — Figure S1: Geographic distribution of ant colonies measured for metabolic rate in this study. Figure S2: Phylogenetic tree of the analyzed ant species. Figure S3: Interspecific colony metabolic scaling of ants, with data for fungus‐farming species mixing the ant and fungus part of colonies (left; n = 53 species) and separating them (right; n = 51 species). Table S1: Phylogenetic correlations (and their respective p values) among ant species traits used as predictors in the analyses of colony‐level metabolic scaling. [file JANE-94-1285-s001.docx]

**Supporting Information**

*Divergent evolution of colony-level metabolic scaling in ants*


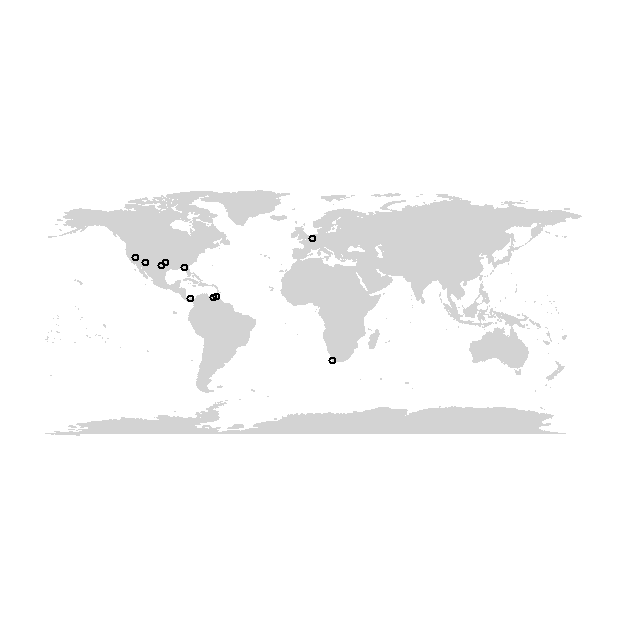
 Figure S1. Geographic distribution of ant colonies measured for metabolic rate in this study. Each circle represents one sampling site.


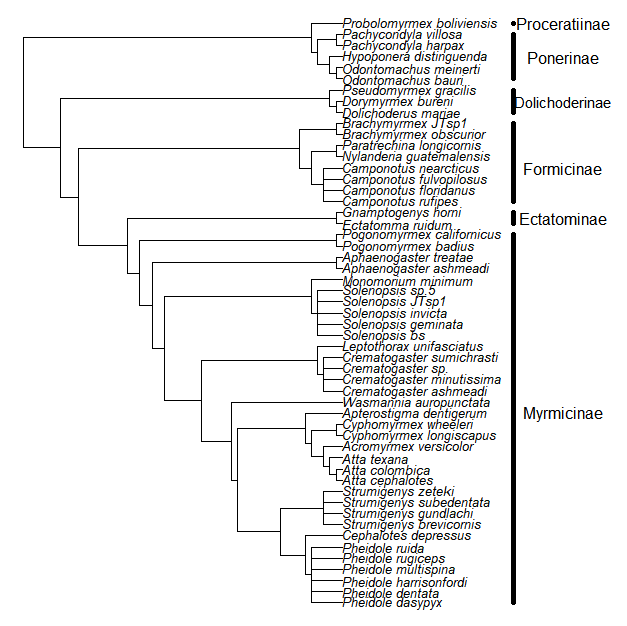


Figure S2. Phylogenetic tree of the analyzed ant species. The tree was created using the genus-level supertree from AntWiki (2024) as backbone and representing within-genus relationships as polytomies. Species names were checked for current validity and updated as required, also according to AntWiki (2024). Branch lengths are arbitrary.


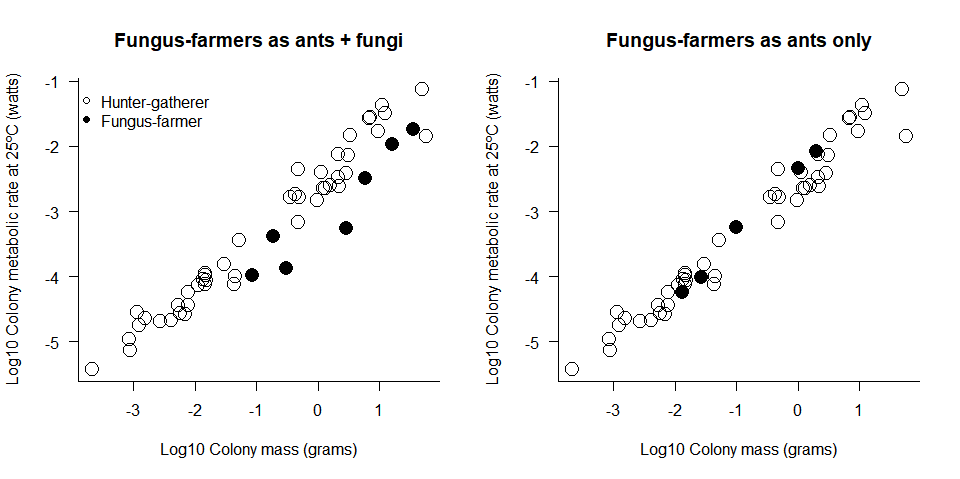


Figure S3. Interspecific colony metabolic scaling of ants, with data for fungus-farming species mixing the ant and fungus part of colonies (left; n = 53 species) and separating them (right; n = 51 species).

Table S1. Phylogenetic correlations (and their respective *P* values) among ant species traits used as predictors in the analyses of colony-level metabolic scaling. Correlations accounted for phylogenetic autocorrelation according to a Brownian model. Mass: colony mass (g). Trophic: trophic level (range: 1.66 to 4.21). Foraging: foraging coordination level (ordinal variable with five ranks, from solitary to trunk-trail). Bold numbers are statistically supported (*P* < 0.05).

|  | Mass | Trophic | Foraging |
| --- | --- | --- | --- |
| Trophic | **-0.35 (0.010)** | - | - |
| Foraging | -0.12 (0.406) | 0.03 (0.834) | - |
| Polymorphism | **0.50 (0.000)** | **-0.28 (0.039)** | 0.06 (0.672) |
